# Potency analysis of cellular therapies: the emerging role of molecular assays

**DOI:** 10.1186/1479-5876-5-24

**Published:** 2007-05-30

**Authors:** David F Stroncek, Ping Jin, Ena Wang, Betsy Jett

**Affiliations:** 1 Department of Transfusion Medicine, Clinical Center, National Institutes of Health, Bethesda MD, 20892, USA

## Abstract

Potency testing is an important part of the evaluation of cellular therapy products. Potency assays are quantitative measures of a product-specific biological activity that is linked to a relevant biological property and, ideally, a product's *in vivo *mechanism of action. Both *in vivo *and *in vitro *assays can be used for potency testing. Since there is often a limited period of time between the completion of production and the release from the laboratory for administration to the patient, *in vitro *assays such are flow cytometry, ELISA, and cytotoxicity are typically used. Better potency assays are needed to assess the complex and multiple functions of cellular therapy products, some of which are not well understood. Gene expression profiling using microarray technology has been widely and effectively used to assess changes of cells in response to stimuli and to classify cancers. Preliminary studies have shown that the expression of noncoding microRNA which play an important role in cellular development, differentiation, metabolism and signal transduction can distinguish different types of stem cells and leukocytes. Both gene and microRNA expression profiling have the potential to be important tools for testing the potency of cellular therapies. Potency testing, the complexities associated with potency testing of cellular therapies, and the potential role of gene and microRNA expression microarrays in potency testing of cellular therapies is discussed.

## Background

Cellular therapies are making a major contribution to the emerging field of biologic therapy. The possibilities for the clinical application of new cellular therapy products are expanding rapidly as is their clinical promise. The diversity and effectiveness of cellular therapies that are now available has encouraged the development of new clinical applications and improved the quality of life of patients. These therapies include adoptive immune therapy utilizing enriched or *in vitro *manipulated autologous or allogeneic immune cells to treat cancer and viral infections [1,2]; β islet cell transplantation [3], hematopoietic progenitor cells (HPC) for transplantation; HPC therapy for cardiac ischemia [4,5], and gene therapy [6]. As this field matures, the ability to produce large quantities of biological products with predictable quality and quantifiable potency is becoming critical.

The complexity of cellular therapies is also increasing as new knowledge about the function of specific cell types and their biologic status becomes available. For example, the initial adoptive immune therapy protocols to treat cancer once only involved the administration of autologous tumor infiltrating leukocytes (TIL) [7] or leukocyte activated killer cells (LAK) [8]. Now adoptive immune therapy protocols are combination therapies that include high dose chemotherapy, the administration of *in vitro *activated and primed TIL, and autologous HPCs [9]. Immunosuppressive chemotherapy depletes the patient's naturally occurring repertoire of lymphocytes including T regulatory cells. The lack of T regulatory cells and increased levels of cytokines, including IL-7, that are associated with leukopenia allow for the rapid and marked *in vivo *expansion of TIL administered with hematopoietic progenitor cells HPCs [9].

Similarly, HPC transplants have become more complex. While hematopoietic progenitor cell transplants (HPCTs) have been used successfully to treat leukemia for more than 30 years, this therapy has been constantly evolving. These changes involve tailoring and optimizing its efficacy by using HPC transplantation in combination with immune therapy to treat leukemia. Either manipulated or unmanipulated lymphocytes from HCPT donors are often administered to recipients following transplantation to prevent or treat disease relapse and enhance immune recovery [10,11].

The sources of hematopoietic progenitor cells used for transplantation have also changed. Early HCPTs were performed exclusively using bone marrow as a source of progenitors. Now, in addition to bone marrow, G-CSF-mobilized progenitors collected from the peripheral blood by apheresis and umbilical cord blood (UCB) are used for HPCT.

Many clinical cellular therapy products require cell mobilization, collection, subset isolation, *in vitro *or *in vivo *stimulation, and culture of cells over a period of several days. The production of some cellular therapies involves serial isolation steps and multiple stimulation and/or culturing steps. Cellular therapy product manufacture is further complicated by donor or patient genetic and physiological heterogeneity. The final product is often markedly different from the starting material. Because of the complex nature of producing cellular therapy products and the clinical importance of the final products, most institutes conducting cellular therapy have developed specialized good manufactory product (GMP) laboratories devoted to the production of these therapeutic agents. The goal of these cell processing laboratories is to produce cellular therapy products that provide the desired clinical affect without resulting in adverse effects. These specialized laboratories ensure that an adequate dose of cells is provided to each patient, each product meets release specifications, and lot-to-lot variation is minimized. In order to produce consistently high quality products, quality assurance has become a critical part of cellular therapy laboratories.

All cellular therapy products must be demonstrated to be safe, pure, potent, stable, and effective for human use. Objective standards based on clinical trial and manufacturing data should be established to evaluate safety and quality characteristics of clinical products during production and at the time of lot release. Also known as product specifications, these standards are intended to ensure that cellular products consistently meet regulatory and industry requirements for sterility, safety, purity, identity, and potency. Tests to measure and evaluate these parameters are performed at critical steps in the manufacturing process (in-process testing) and at the end of the production prior to the release of the product for clinical use (lot release testing). The results of in-process and lot release assays should fall within specified ranges and meet predetermined acceptance criteria before the product can be released for human use. In-process testing and lot release testing are important for assuring individual product quality as well as lot-to-lot consistency. For cellular therapies, these assays include tests of sterility, including mycoplasma, viability, and assessment of product potency. One of the most important aspects of assessing the quality of cellular therapy products is to ensure that all products meet established minimal levels or ranges of potency and that potency levels are consistent across manufacturing lots.

### Potency testing

Potency testing involves the quantitative measure of biological activity of a product. The biological activity describes the ability of a product to achieve a defined biological effect. Potency testing is the quantitative measure of a biological activity which is linked to relevant biological properties of a product. The biological activity measured should be closely related to the product's intended biological effect and ideally it should be related to the product's clinical response [12-14].

Potency assessments are meant to measure a cellular therapy product's critical biological activity within a complex mixture by quantifying the product's activity in a biological system. Measurement of the potency of a product is not the same as measuring clinical efficacy, but rather a means to control product consistency. Generally, potency testing is performed at the time of product lot release and across all production lots.

Since potency assays for cellular products usually take a considerable amount of time to develop, generally, the development of potency assays is progressive. The development of potency assays usually begins during preclinical and early clinical development. Development starts with identifying the critical biological activity of the product and formulation of an approach to potency determination. A potency assay should be validated prior to phase III clinical trails [12,13].

### Complexities associated with potency testing of cellular therapies

Potency testing of cellular therapies is particularly challenging for several reasons (Table 1). First of all, since most cellular therapies are patient-specific, there is usually a limited quantity of suitable source material and, therefore, a limited amount of final ready to administer biologic material to use for lot release and potency testing. The starting materials for most cellular therapies are cells collected from human subjects. The subjects may be the person being treated, autologous products, or a living donor, allogeneic products. For both situations the quantity of starting material that can be collected is limited and consequently the amount of material produced is limited. As a result an entire production lot of a cellular therapy is usually administered to a single patient and the use of large quantities of the product for lot release testing may adversely affect the dose and clinical effectiveness of the product. This limitation on the quantity of material available prevents the use of some assays and/or limits the number of analytes that can be tested.

**Table 1:** Challenges associated with potency testing of cellular therapies

▪Limited quantity of final product to test
▪Time to perform lot release testing is usually limited
▪Stability of most cellular therapy products is limited
▪Limited availability of reference standards
▪Variability among lots is generally very high

Second, the time to test the product is limited since cellular therapy products must be tested at the time production is complete, but prior to being released for clinical use. This is particularly problematic for cellular therapies since the potency of many living cells is affected by prolonged storage at physiological temperatures. In fact, some products must be administered within hours upon production completion. In addition, handling affects the potency of some products.

Most potency assays require reference preparations with an established potency which are used as assay standards [12]. The limited availability of reference standards complicates potency testing for cellular therapies. Often "in-house" reference standards must be developed. When reference standard are commercially available, they are may be expensive.

Finally, cellular therapy products typically show a large degree of lot-to-lot variability. Product variability is due in part to inherent variability in the starting cells or tissues. Donor genetic factors likely contribute to differences in potency of the final cellular therapy product. Genetic polymorphisms in cytokines, growth factors and their receptors affect the cellular immune response [15-18]. It is likely that these polymorphisms affect the response of cells to cytokine and growth factor stimulation *in vitro *and the behavior of cells during culture. Epigenetic changes may also be important. The same type of cells obtained from different donors at different time points and under different physiological conditions could vary significantly due to genetic heterogeneities, epigenetic differences, or transcription regulation diversities.

### Factors affecting the potency of cellular therapies

Despite the difficulties associated with potency testing of cellular therapies, potency is particularly important for these products since the complexities associated with their production can result in considerable differences in potency among different lots of the same product (Table 2). These differences are related to the multiple steps required to produce most cellular therapies, variations in starting materials, limited stability of the final product, complex mechanisms of action of the product, and genetic differences among individuals donating the starting cells.

**Table 2:** Factors contributing to the complex nature of cellular therapies

▪Variations in the starting cellular material
▪Multiple biological products may be used in the manufacturing process
▪Multiple steps can be involved in the manufacturing process
▪Clinical effectiveness may be dependent on multiple cellular functions

Advanced cellular therapies may incorporate multiple components. For example, cellular products used for cancer vaccines may require more than one peptide to educate immune cells *in vitro*, followed by cytokine stimulation. A manipulated lymphocyte component prepared for a HPCT donor may involve isolating and recombining multiple different types of cells. The multiple cell types present in many cellular therapies have the potential to interfere with one another or to act synergistically.

Many cellular therapies are subject to extensive manipulation, including manufacturing processes such as cytokine, growth factor or antigen stimulation; culture; expansion; and treatment with vectors or toxins. For these products, slight variations in the starting cellular material, reagents, processing methods, or culture conditions may result in significant variation in the final product leading to heterogeneous clinical out comes of the same therapies.

Finally, the *in vivo *function of most cellular therapies is dependent on multiple factors in the host environment. Hematopoietic stem cells must traffic to specific sites, expand, and differentiate into several mature cell types. Immune therapies must migrate from the site of administration, interact with tumor or other immune cells, and respond to stimuli and/or stimulate other cells.

### Measuring potency of cellular therapies

Potency can be tested in a number of ways including *in vivo *and *in vitro *systems (Table 3). Testing potency using *in vivo *animal models is generally preferred over *in vitro *test systems since animal models assays have the ability to directly measure a product's functional activity. However, existing animal models may not be relevant and new animal models may be difficult to develop [12]. In addition, the results of *in vivo *tests are often variable and difficult to reproduce. Furthermore, these assays usually take a considerable amount of time to complete making it difficult to use these assays for routine lot release testing. Many *in vivo *assays are best suited for use in product development, as an in-process control, or to evaluate the potential effect of changes in the manufacturing process or materials [13].

**Table 3:** Assays used for potency testing

***In vivo *assays**
▪Animal models
***In vitro *assays**

▪Cell-based assay systems
▪ELISA
▪Flow cytometry
▪ELISPOT
▪Proteomics
▪Quantitative real-time PCR

*In vitro *assays involve the measurement of biochemical or physiological responses at the cellular level [12]. The *in vitro *measurement of cell surface markers, activation markers, secretion of factors, or protein expression do not directly measure the function of a cellular product, however, they have been used as surrogates for potency. When an *in vitro *assay is used as a surrogate for potency, a correlation should be demonstrated between the assay results and the intended biological activity. Typical *in vitro *assays used as surrogates for potency testing include ELISA, ELISPOT, flow cytometry, proteomic analysis and cytotoxicity assays.

When the mechanism of action of a cellular therapy can be attributed to the expression of specific cell surface antigens, the measurement of antigens by flow cytometry can be used as an *in vitro *potency assay. In fact, the measurement of biomarkers by flow cytometry is often used as a surrogate measure of cell potency. Flow cytometry is useful due to the large number of reagents and assays that are available as well as the relatively quick turn around time. It can be used to measure the expression of cell surface markers, viability, and the production of cytokines. Extensive analysis of cell surface markers using flow cytometry has been used to assess cellular therapies, but the maximum number of makers that can be analyzed is limited by the availability of specific antibodies, instrumental detection limits, and final product quantity. In addition, the markers that maybe most useful may not be known

*In vitro *cell function assays have also been used to measure cell potency. Cytotoxicity assays are sometimes used to reflect the function of adoptive immune therapies. Cytokine release by stimulated cells can also be used to measure cell function. However, cell function assays have many limitations. While they maybe able detect differences in relevant biological activity, these assays are typically highly specialized for each cell type, labor intensive, and require highly skilled staff. Different types of cells and cell subsets require completely different types of cell function technologies. Many cells require the measurement of multiple functions to adequately assess potency. Furthermore, the function(s) which best predict cell potency may not be known. In fact, for many cellular therapies, all aspects that contribute to *in vivo *activity are not completely understood. In addition to these limitations, many cell processing laboratories working with cellular therapies in phase I and II clinical trials prepare several different types of cellular therapies. It is possible, but may not always be feasible for a centralized cell processing laboratory to perform several different types of cell function assays.

Cell counts and viability measurements are often preformed on cellular therapies. However, since these assays do not measure a relevant biological activity, they are not potency measures [12,13].

### Gene expression microarrays for potency testing

Measurements of the expression of genes related to a specific cellular activity or function could be used as an *in vitro *biomarker of potency. Quantitative real-time PCR assays are useful tools for assessing the expression of individual genes in order to assess the activity of immune cells. The measurement of changes in interferon gamma transcription by quantitative real-time PCR has been used to as a marker for T cell activation following stimulation with a recall antigen [19-22]. Quantitative real-time PCR has recently been used to measure the production of mRNA encoding interferon-γ, interleukin-2 (IL-2), IL-4 and IL-10 by stimulated T cells [20]. Quantitative real-time PCR arrays are also available to assess angiogenesis, apoptosis, cell cycle, insulin signaling pathways, cytokines and receptors, nitric oxide signaling pathways and JAK/STAT signaling pathways.

While using quantitative real-time PCR to measure the expression of single genes or groups of genes is helpful in assessing cell function, the complete assessment of the function of cellular therapies requires the measurement of a broad range of gene transcripts, especially when the mechanisms responsible for effective therapy are not thoroughly understood. The analysis of cells using gene expression microarrays allows the simultaneous assessment of the expression of thousands of genes. One practical advantage of gene expression microarray assays over other analytical assays is that very few cells are needed. Enough RNA can be isolated from 1 × 10^4 ^to 1 × 10^6 ^cells for analysis with a 17,500 gene cDNA expression microarray [23].

Microarrays with 15,000 to 40,000 genes or oligonucleotide probes have been used clinically to characterize lymphomas [24], prostate cancer [25], ovarian cancer [26], small cell lung cancer [27], and melanoma [28] and many other cancers. We have used cDNA gene expression microarrays with 17,500 genes to investigate the immunologic changes associated with high dose IL-2 therapy for renal cell carcinoma [29] and imiquimod, a TLR-7 ligand, therapy for basal cell carcinoma [30]. We have also used cDNA microarrays to assess the effects of IL-10 on NK cells [31-33] and several different types of interferon on LPS-stimulated mononuclear cells, the *in vitro *response of mononuclear cells to IL-2 [34], and the molecular basis of cutaneous wound healing [35].

While gene expression microarrays have been widely used to assess changes in cells in response to stimuli, or to classify different types of cancers, they have only been used to a limited extent to assess cell potency. However, since gene expression microarrays simultaneously measure the expression of thousands of genes, they capture a snap shot of all possible gene expression signatures which are associated with cellular function and hence could be a very important tool for assessing the potency of cellular therapies. The comprehensive nature of gene expression microarray analysis makes them ideal for measuring both expected and unexpected cell functions. This is particularly important for the analysis of cells with complex and multiple critical functions such as dendritic cells (DCs), embryonic stem cells, and hematopoietic stem cells.

In addition to assessing potency, gene expression microarrays can also assess other important aspects of cellular therapy products such as stability, purity, maturation and differentiation status. Since microarrays can detect the activation of apoptosis pathways that signal the onset of cell death, they have the potential to provide useful information concerning the effects of storage or manipulation on cell viability. The assessment of the expression of apoptosis genes is likely to be much more sensitive in assessing cell viability than dye exclusion assays or the flow cytometric measurement of Fas or annexin. Gene expression profiles can also detect subpopulation of cells and therefore provide information concerning cell purity.

There are some limitations concerning the use of gene expression microarrays for potency testing. Gene expression microarray analysis involves multiple steps including RNA isolation, amplification, fluorescent labeling, hybridization and data analysis. It is impossible at the current technology stage to complete the whole procedure within a few hours and so these global expression microarrays can not yet be used for lot release testing. However, if global microarrays can identify specific sets of gene whose expression is associated with potency, tailored chips or quantitative real-time PCR kits which only assess specific "potency genes" could be developed and used for lot release testing.

### Potential applications of gene expression profiling for potency testing

#### Predicting the confluence of human embryonic kidney 293 cells

Gene expression microarrays have been demonstrated to be useful for some cell therapy applications. They can be used to predict the quality of cells used to manufacture biologic products. Human embryonic kidney (HEK) 293 cells are often used to manufacture products such as adenoviral gene therapy vectors and vaccines [36]. These cells can be grown in bioreactors, tissue culture flasks, and roller bottles. However, when HEK 293 cells grow to form a confluent monolayer, their phenotype changes as does the quality of the vector or vaccine produced by these cells. Cell confluence can be readily assessed by visual inspection of cells grown in flasks and roller bottles, but for cells grown in bioreactors, the assessment of confluence by visual inspection is not always possible. Gene expression profiling has been used to identify genes whose expression predicts cell confluence [36]. Human embryonic kidney 293 cells that have been grown to 90% confluence have a unique gene expression signature compared to those grown to 40% confluence. A set of 37 of these signature genes is able to predict that quality and confluence of HEK 293 cells. While this use of gene expression profiling does not represent a potency assay, it demonstrates the potential of the use of gene expression profile assays.

#### Cell differentiation status analysis of embryonic stem cells

Human embryonic stem cells (hESC) have the potential to be useful for a number of clinical applications. Since cultured hESC may undergo spontaneous differentiation, it is important to determine if cultured hESC have maintained their stem cell qualities or if they have begun to acquire properties of more differentiated cells. Gene expression profiling may be useful for assessing cultured hESC. Gene expression profiling has been used to identify genes that are uniquely expressed by hESC [37]. Player et al have found that 1715 genes were differentially expressed between hESC and differentiated embryonic cells [37]. The analysis of the expression of genes that are expressed by hESC but not by differentiated cells is likely to be useful in determining if cells in culture have maintained their embryonic stem cell characteristics.

Embryonic stem cells must be differentiated before they can be used clinically. One of the first steps in the differentiation of hESC into mature cells and tissues for clinical use is the production of embryoid bodies (hEB). The production of embryiod bodies involves the aggregation of embryonic cells but the prevention of separation of cells into germ lines by plating them onto a non-permissive substrate. After these hEB are isolated they can be induced to generate several different types of cells including hematopoietic cells, neuronal, myogenic, and cardiac muscle cells. A comparison of genes expression profiles of hESC and hEB has found that the expression of several genes were down regulated and several were upregulated including 194 whose expression was more than 3-fold greater in hEB [38]. This unique set of genes should also be useful in assessing hESC differentiation.

#### Potency testing of hematopoietic progenitors

While hESC are not yet being used for clinical applications, hematopoietic stem cells are widely used for several clinical applications and better potency assays for these therapies are needed. Potency assays for hematopoietic stem cell products used for transplantation should measure the ability of the product to reconstitute bone marrow hematopoietic cells and peripheral blood cells in the transplant recipient. The potency assay should reflect the period of time that neutrophil, platelet, and red blood cells counts return to and remain above specified levels independent of transfusion therapy. In another words if the potency assay indicates that a product meets minimum criteria, the therapy should result in at least minimum acceptable neutrophil, platelet, and RBC counts in the recipient for a minimum specified duration of time.

Liquid culture of long-term culture initiating cells (LTC-IC) and the repopulation of marrow in nonobese diabetic (NOD)/severe combined immunodeficiency (SCID) mice assays are considered to be the best measure of the quantity and quality of hematopoietic stem cells. However, these assays require several weeks to complete, highly specialized reagents, and highly trained staff. As a result these assays have seldom, if ever, been used as potency assays.

The measurement of myeloid, erythroid, and mixed colony formation in methylcellulose culture systems has been the standard method for assessing bone marrow and PBSC concentrates, but they have been used mainly as in-process controls. The measurement of colony formation in methyl cellulose is an effective biological assay that directly measures a relevant function of HPCs, however, these assays take approximately 14 days to complete and consequently they can not be used as a potency assay.

Traditionally, total nucleated cells counts were used to assess the potency of bone marrow and are still used as a measure of potency of UCB components prepared for transplantation. Regulations suggest that UCB components contain ≥ 90 × 10^7 ^total nucleated cells including nucleated RBC and that ≥ 85% of nucleated cells are viable [39]. However, the measurement of CD34+ cells by flow cytometry, has become the universal assay for measuring the potency of HPC products collected by apheresis from subjects treated with hematopoietic growth factors. The number of CD34+ cells in a HPC product can be measured within a few hours using anti-CD34 and flow cytometry and this assay is well-suited for lot release testing. Generally, a dose of 1 × 10^6 ^per kg of CD34+ G-CSF-mobilized peripheral blood stem cells (PBSCs) is considered adequate for an autologous transplant and 3 to 5 × 10^6 ^CD34+ cells per kg for an allogeneic transplant. Umbilical cord blood components must on contain ≥ 1.25 × 10^6 ^viable CD34+ cells.

While CD34 antigen expression is widely used as a measure of potency of HPCs collected from the peripheral blood, HPCs expressing CD34 antigen do not represent a homogenous population. Several distinct subpopulations or phenotypes of CD34+ cells have been described [40]. Some subpopulations are more primitive, while others are more likely to different into myeloid cells, erythroid cells or megakaryocytes.

Despite the heterogeneity of CD34+ cells, the measurement of CD34+ cells has been an effective measurement of potency of PBSC concentrates collected by apheresis. This is likely because PBSC components are relatively similar in that almost all PBSC components are collected from subjects given granulocyte colony-stimulating factor (G-CSF) alone or in combination with chemotherapy. However, the sources of stem cells and types of mobilizing agents used for transplantation are changing. UCB components are being used in place of PBSC concentrates and marrow for unrelated donor HPC transplantation. A new stem cell mobilizing agent, AMD3100, is being used with G-CSF to mobilize stem cells for autologous transplants [41] and will likely soon be used for allogeneic donor transplants [42,43]. CD34+ cells from both UCB and AMD3100-mobilized PBSC concentrates differ from those found in G-CSF-mobilized PBSC concentrates and the quantity of CD34+ required for a successful transplant from some of these types of products will likely differ from the quantity required for a successful G-CSF-mobilized PBSC transplant.

AMD3100 mobilizes stem cells by a different mechanism than G-CSF. AMD3100 is a CXCR4 antagonist and it mobiles stem cells within 6 hours by disrupting the binding of stem cell CXCR4 with SDF-1, CXCL12, on marrow osteoblasts [44]. In contrast G-CSF mobilizes stem cells indirectly by down regulating the expression of SDF-1 on marrow osteobasts and by releasing neutrophil and monocyte proteolytic enzymes including neutrophil elastase, cathepsin G, and maxtrix metalloproteinase-9 that degrade important HPC trafficking and adhesion molecules c-kit, VCAM-1, CXCR4, and SDF-1 [44]. Because of the differences in mechanisms of mobilization between AMD3100 and G-CSF, AMD3100 mobilizes a CD34+ cell population with a greater long-term marrow repopulating capacity and with a different phenotype than G-CSF [43].

The potency of UCB CD34+ cells also differs from that of G-CSF-mobilized peripheral blood CD34+ cells. The potency of CD34+ cells from UCB as measured by the ability to repopulate NOD SICD mice is greater than the potency of CD34+ cells from bone marrow or G-CSF mobilized PBSCs [45-47]. In addition, UCB CD34+ cells show increased proliferative capacity compared to bone marrow and G-CSF-mobilized PBSC CD34+ cells in methycellulose culture [47-49].

Since the potency of CD34+ cells is dependent on the number of and subtypes of CD34+ cells and since the sources of hematopoietic progenitor cells used in transplantation is increasing, new potency assays are needed. Preliminary comparison of CD34+ cells mobilized by G-CSF and G-CSF plus AMD3100 using gene expression profiling has identified 81 genes whose expression in 3 subjects was increased in G-CSF plus AMD-3100 mobilized CD34+ cells and 29 genes whose expression was decreased [50]. Genes whose expression was increased included those involved with anti-apoptosis, cell cycle, replication/DNA repair, cell mobilization and oxygen transport. Further work is needed to identify HPC genes whose expression best correlates with the results of traditional potency assays such as colony formation assays.

#### Potency testing of dendritic cells

Dendritic cells (DCs) are potent professional antigen presenting cells capable of capturing and processing antigens in order to present peptides to prime T cells [51]. They express both HLA class I and class II molecules and present peptides to CD4+ and CD8+ T cells. They also express co-stimulatory molecules such as CD80, CD86, CD40, ICAM-1, and LFA-3. For immune therapy, DCs can be generated from PBMCs after GM-CSF and IL-4 stimulation *in vitro*, or they can be generated by co-culturing *in vitro *with irradiated tumor cells or virus infected cells, proteins or peptides. Mature DCs are then administered to patients to stimulate cytotoxic T cells *in vivo*. Immunotherapies with DCs are being used to treat melanoma, renal cell carcinoma, prostate cancer and leukemia [51].

Since few DCs are present in the blood, they must be produced from other types of cells. DCs for clinical therapies produced from CD34+ cells are known as plasmacytoid DCs and those produced from circulating mononuclear cells are know as myeloid-derived DCs. Either mature or immature DCs can be produced. Immature DCs express lower levels of HLA class II antigens and lower levels of co-stimulatory molecules but higher levels of Fc and mannose receptors. The ability of immature DCs to phagocytosize and process antigens is better than that of mature DCs, but mature DCs present antigens better than immature DCs. While the function of mature and immature DCs differ, it is not possible with standard analytic assays to precisely distinguish the degree of maturation of DCs.

The potency of DCs can be tested by assessing the ability of DCs loaded with antigen to stimulate autologous T cells [52]. However, this is difficult because of the low percentage of T cells in most patients that are responsive to tumor antigens. One alternative to overcome the low number of autologous T cells is to generate and expand T cell clones that respond to specific antigens. Even so, only T cells with the same HLA restriction elements and antigen specificity could be used in a DC potency assay. For example, HLA-A*0201 T cell clones specific to a melanoma antigen such as Mart I would not be useful for testing dendritic cells prepared from subjects with other HLA types such as HLA-A*03 or other antigens such as cytomegalovirus (CMV) pp65. Consequently, separate clones must be developed for each antigen and HLA restriction being studied.

The potency of DCs can be assessed by using test peptides from recall antigens that are able to stimulate memory T cell responses [52]. These antigens include HLA-restricted tetanus toxin, influenza virus, and Epstein-Barr virus (EBV) antigens since most people have been immunized against these antigens. However, assays using recall antigens do not directly test DCs ability to present tumor-associated antigens and efficacy to stimulate tumor-specific T cells. So these assays can not be used as a lot release test for DCs used for cancer therapy, although testing the ability of DCs to present recall antigens and stimulate T cells is useful as an in-process control.

The measurement of DC co-stimulatory activity has been used to measure the potency of DCs. Co-stimulation plays a critical role in the induction of antigen-specific immunity. One method to measure co-stimulation is the mixed lymphocyte culture reaction that is based on the stimulation of responder cells with replication competent allogeneic DC stimulator cells. However, it is not known to what degree allo-reactivity and co-stimulation contribute to T cell stimulation.

Alternatively, gene expression profiling is likely to be useful in assessing the potency of DCs used for clinical therapies. It has been used to characterize the differentiation of monocytes into macrophages and their polarization to macrophages with a type 1 or type 2 phenotype [53] and has also been used to characterize the response of monocytes to LPS and cytokine stimulation [32,33]. Preliminary data in our laboratory has also found that gene expression profiling can distinguish monocytes from immature DCs and immature DCs from mature DCs. The ability of gene expression microarrays to assess cells globally may allow them to determine the potency of DCs by evaluating unstimulated cells or cells that have been stimulated with a recall antigen. However, genes whose expression reflects DC maturation as well as specific DC functions must be identified before gene expression profiling can be used as a potency assay for DCs.

### MicroRNAs as potency assays

MicroRNAs (miRNA) are likely to be another important indicator of hematopietic and immune cell potency. miRNAs are an abundant class of endogenous non-protein-coding small RNAs of 19 to 23 nucleotides which are derived from pre-miRNA of 60 to 120 nucleotides. Mature miRNAs negatively regulate gene expression at the post transcriptional level. They reduce the levels of target transcripts as well as the amount of protein encoded. 541 human miRNAs have been so far identified [54]. In general, miRNAs are phylogenetically conserved and, therefore, have conserved and defined post transcription inhibition function. Some miRNAs are expressed throughout an organism, but most are developmentally expressed or are tissue-specific.

MicroRNAs play an important role in many cellular development and metabolic processes including developmental timing, signal transduction, tissue differentiation, and cell maintenance. Most miRNAs are tissue specific. For example the expression of miR-1 is restricted to the heart [55] and miR-223 to granulocytes and macrophages [56]. Recently, miRNA have been found to have a role in stem cell self renewal and differentiation. Several different miRNAs are involved with the differentiation of hematopoietic progenitor cells. MiR-155 is important in preventing the differentiation of CD34+ cells toward myeloid and erythroid cells [57]. In addition, miR-221 and miR-222 prevent the differentiation of hematopoietic stem cells into erythroid progenitors [58]. MiR-181 is involved in the control of lymphopoiesis [56].

MicroRNA seems ideally suited for distinguishing primitive from committed hematopoietic, embryonic and other stem cells as well as different types of lymphocytes and mononuclear phagocytes. However, they have not been evaluated to determine if they would be useful in this capacity. MicroRNA profiles of mononuclear phagocytes and dendritic cells have not been studied extensively, but if miRNA profiles differ between immature and mature DCs, they may be useful in assessing the potency of DCs produced *in vitro*.

The high throughput analysis of miRNAs requires at least 10 times greater quantities of cells than gene expression profiling since miRNAs contribute only about 1% of a cell's total mRNA. MiRNA amplification methods have not yet been fully validated and, hence, are not considered reliable. However, targeted miRNA analysis requires a relatively small number of cells, 1 × 10^6^.

An advantage of miRNA expression profiling compared to gene expression profiling is that miRNA expression profiling requires smaller arrays and chips which make it possible to analyze multiple samples on the same slides containing sub-arrays. While gene expression cDNA microarrays contain 10,000 to 35,000 probes, the number of miRNA currently identified is only in the hundreds.

## Conclusion

As more and more new cellular therapies are being developed and used to treat an increasing variety of diseases and patients, potency testing is becoming a critical and required part of the production of cellular therapies. Existing assays, such as function, flow cytometry, and ELISA are important but limited by the number of factors analyzed. Gene expression microarrays have the potential to become important in potency testing. They are well suited for the assessment of the potency of cellular therapies in phase I and II clinical trials. As data is collected during clinical trials the results of analysis with the gene expression microarrays should be compared with the results of traditional function assays and genes whose expression is associated with critical biological function identified and used to develop assays to rapidly measure the expression of genes associated with cell potency. While it is also worth assessing cellular therapies in phase I and II clinical trials with miRNA expression microarrays, the role of miRNA profile analysis in assessing potency is yet to be tested.
